# Supplementary material for: Polyketide synthase gene clusters in the ericoid mycorrhizal fungus Oidiodendron maius: genome-wide prediction, expression profiling, and functional characterization of knockout mutants
Source: Front Plant Sci. 2026 Sep 16;17:1924430. doi: 10.3389/fpls.2026.1924430 (PMC13623903; doi:10.3389/fpls.2026.1924430)
Supplement: Supplementary file 1 [file SupplementaryFile1.zip › Supplementary Materials/Supplementary Folder 1.zip!/Oidma1_AssemblyScaffolds_Repeatmasked/knownclusterblast/cluster3/jgi.p_Oidma1_99363_mibig_hits.html]

| MIBiG Protein | Description | MIBiG Cluster | MiBiG Product | % ID | % Coverage | BLAST Score | E-value |
| --- | --- | --- | --- | --- | --- | --- | --- |
| BAC20568.1 | efflux\_pump | BGC0000039 | Compactin\_biosynthetic\_gene\_cluster | 61 | 91.8003565062 | 615 | 7e-176 |
| ABA02247.1 | efflux\_pump | BGC0000098 | Monacolin\_K\_biosynthetic\_gene\_cluster | 59 | 90.5525846702 | 585 | 7.8e-167 |
| AAD34558.1 | unknown | BGC0000089 | Lovastatin\_biosynthetic\_gene\_cluster | 59 | 90.1960784314 | 584 | 1e-166 |
| CAP96439.1 |  | BGC0000420 | Roquefortine\_C\_/\_meleagrin\_biosynthetic\_gene\_cluster | 41 | 89.1265597148 | 381 | 2.2e-105 |
| ADY16699.1 | TqaJ | BGC0001142 | Tryptoquialanine\_biosynthetic\_gene\_cluster | 40 | 93.4046345811 | 370 | 3e-102 |
| EAQ86390.1 | hypothetical\_protein | BGC0001405 | Chaetoviridin\_/\_chaetomugilin\_biosynthetic\_gene\_cluster | 44 | 76.4705882353 | 367 | 3.3e-101 |
| AAS90069.1 | AflT | BGC0000010 | Aflatoxin\_biosynthetic\_gene\_cluster | 40 | 95.5436720143 | 366 | 4.4e-101 |
| AGN71625.1 | putative\_HC-toxin\_efflux\_carrier\_TOXA | BGC0000027 | Azaphilone\_biosynthetic\_gene\_cluster | 55 | 58.6452762923 | 365 | 1.3e-100 |
| AMR44276.1 | MFS\_multidrug\_transporter | BGC0001398\_c3 | Phomopsins\_biosynthetic\_gene\_cluster | 40 | 93.9393939394 | 364 | 2.8e-100 |
| AAS90092.1 | AflT | BGC0000006 | Aflatoxin\_biosynthetic\_gene\_cluster | 40 | 95.7219251337 | 363 | 6.3e-100 |
| AAS90021.1 | AflT | BGC0000008 | Aflatoxin\_biosynthetic\_gene\_cluster | 40 | 95.7219251337 | 361 | 1.8e-99 |
| XP\_002384861.1 | MFS\_multidrug\_transporter,\_putative | BGC0001234\_c2 | 4,4'-piperazine-2,5-diyldimethyl-bis-phenol\_biosynthetic\_gene | 39 | 84.8484848485 | 360 | 3.1e-99 |
| AAS89998.1 | AflT | BGC0000007 | Aflatoxin\_biosynthetic\_gene\_cluster | 41 | 89.8395721925 | 358 | 1.5e-98 |
| AAS90046.1 | AflT | BGC0000009 | Aflatoxin\_biosynthetic\_gene\_cluster | 40 | 97.6827094474 | 356 | 5.9e-98 |
| BAD29973.1 | transporter\_protein | BGC0000676 | Aphidicolin\_biosynthetic\_gene\_cluster | 38 | 89.3048128342 | 344 | 1.8e-94 |
| BAE71313.1 | putative\_ABC\_transporter | BGC0000004 | Aflatoxin\_biosynthetic\_gene\_cluster | 41 | 81.8181818182 | 342 | 1.1e-93 |
| CBF87265.1 | MFS\_toxin\_efflux\_pump\_(AflT),\_putative\_(AFU\_orthologue;\_AFUA\_1G12620) | BGC0000680\_c2 | Austinol/dehydroaustinol,\_locus\_1\_biosynthetic\_gene\_cluster | 36 | 97.8609625668 | 335 | 1.1e-91 |
| ACD39756.1 | major\_facilitator\_superfamily\_transporter | BGC0000076 | Hypothemycin\_biosynthetic\_gene\_cluster | 37 | 88.7700534759 | 325 | 1.5e-88 |
| ACD39765.1 | major\_facilitator\_superfamily\_transporter | BGC0000077 | Hypothemycin\_biosynthetic\_gene\_cluster | 37 | 88.7700534759 | 325 | 1.5e-88 |
| ADM34144.1 | efflux\_pump | BGC0001084 | Notoamide\_/\_stephacidin\_biosynthetic\_gene\_cluster | 35 | 93.4046345811 | 292 | 8e-79 |
| AHV78251.1 | ResE | BGC0001246 | Trans-resorcylide\_biosynthetic\_gene\_cluster | 34 | 93.76114082 | 290 | 3e-78 |
| AAB36607.1 | toxin\_pump | BGC0000370\_c2 | HC-toxin\_biosynthetic\_gene\_cluster | 33 | 89.3048128342 | 282 | 1.4e-75 |
| CBF76046.1 | conserved\_hypothetical\_protein | BGC0001399 | Fellutamide\_B\_biosynthetic\_gene\_cluster | 36 | 82.174688057 | 281 | 2.4e-75 |
| EAU36749.1 | predicted\_protein | BGC0000292 | Acetylaranotin\_biosynthetic\_gene\_cluster | 36 | 85.9180035651 | 252 | 9.2e-67 |
| ctg1\_orf0002 |  | BGC0000688 | Copalyl\_diphosphate\_biosynthetic\_gene\_cluster | 39 | 67.7361853832 | 245 | 1.9e-64 |
| CAF60521.1 | putative\_efflux\_protein | BGC0000704 | Kanamycin\_biosynthetic\_gene\_cluster | 31 | 85.2049910873 | 212 | 1.4e-54 |
| CAF31575.1 | putative\_kanamycin\_efflux\_protein | BGC0000705 | Kanamycin\_biosynthetic\_gene\_cluster | 31 | 85.2049910873 | 212 | 1.4e-54 |
| BAE56594.1 |  | BGC0001123 | Aspirochlorine\_biosynthetic\_gene\_cluster | 33 | 75.5793226381 | 204 | 3.7e-52 |
| AAF00219.1 | transporter | BGC0000277 | Urdamycin\_biosynthetic\_gene\_cluster | 30 | 90.1960784314 | 197 | 3.5e-50 |
| AHW57792.1 | PgaJ3 | BGC0000262 | Rabelomycin\_biosynthetic\_gene\_cluster | 30 | 80.926916221 | 193 | 8.6e-49 |
| AFU65888.1 | DacR2 | BGC0000216 | Dactylocycline\_biosynthetic\_gene\_cluster | 32 | 81.1051693405 | 188 | 2.8e-47 |
| ACP19369.1 | SaqJ1 | BGC0000267 | Saquayamycin\_Z\_/\_galtamycin\_B\_biosynthetic\_gene\_cluster | 31 | 86.9875222816 | 180 | 4.4e-45 |
| AHA12095.1 | transporter | BGC0001172 | Chlorizidine\_A\_biosynthetic\_gene\_cluster | 30 | 87.1657754011 | 180 | 7.6e-45 |
| BAV16993.1 | putative\_transporter | BGC0001384 | Saprolmycin\_biosynthetic\_gene\_cluster | 29 | 86.9875222816 | 178 | 2.9e-44 |
| AGO50605.1 | transporter | BGC0000229 | Grincamycin\_biosynthetic\_gene\_cluster | 30 | 72.5490196078 | 170 | 6e-42 |
| ADG86335.1 | transporter | BGC0000190 | A-74528\_biosynthetic\_gene\_cluster | 28 | 79.5008912656 | 161 | 2.8e-39 |
| ADE34501.1 | SsfR | BGC0000269 | SF2575\_biosynthetic\_gene\_cluster | 29 | 78.7878787879 | 148 | 3.2e-35 |
| ABC87523.1 | putative\_drug\_efflux\_transporter | BGC0001011 | Meridamycin\_biosynthetic\_gene\_cluster | 28 | 83.422459893 | 143 | 7.8e-34 |
| CAA09636.1 | putative\_transmembrane\_protein | BGC0000227 | Granaticin\_biosynthetic\_gene\_cluster | 28 | 77.3618538324 | 143 | 7.8e-34 |
| AJI44184.1 | drug\_resistance\_transporter\_EmrB/QacA | BGC0001193 | Thiolutin\_biosynthetic\_gene\_cluster | 29 | 69.5187165775 | 131 | 2.4e-30 |
| AAQ08939.1 | putative\_membrane\_transporter | BGC0000224 | Fredericamycin\_biosynthetic\_gene\_cluster | 27 | 78.2531194296 | 120 | 7.1e-27 |
| AGA37273.1 | efflux\_pump | BGC0000819 | Paraherquamide\_biosynthetic\_gene\_cluster | 39 | 35.8288770053 | 119 | 9.3e-27 |
| CAP12594.1 | elloramycin\_permease | BGC0000219 | Elloramycin\_biosynthetic\_gene\_cluster | 27 | 78.7878787879 | 118 | 3.5e-26 |
| ABI22121.1 | transmembrane\_efflux\_protein | BGC0000422 | Saframycin\_A\_biosynthetic\_gene\_cluster | 28 | 74.3315508021 | 116 | 7.9e-26 |
| EHM27501.1 | putative\_export\_protein | BGC0000235 | Kiamycin\_biosynthetic\_gene\_cluster | 27 | 70.944741533 | 116 | 1e-25 |
| AAA67509.1 | tetracenomycin\_C\_resistance\_and\_export\_protein | BGC0000275 | Tetracenomycin\_biosynthetic\_gene\_cluster | 27 | 73.7967914439 | 116 | 1.3e-25 |
| AAR98561.1 | GntO | BGC0000697 | Gentamicin\_biosynthetic\_gene\_cluster | 26 | 75.0445632799 | 112 | 1.9e-24 |
| CAF31444.1 | putative\_gentamicin\_exporter | BGC0000696 | Gentamicin\_biosynthetic\_gene\_cluster | 26 | 75.0445632799 | 112 | 1.9e-24 |
| CAF34033.1 | putative\_transmembrane\_efflux\_protein | BGC0000689 | 2-deoxystreptamine\_biosynthetic\_gene\_cluster | 26 | 75.0445632799 | 112 | 1.9e-24 |
| ACN38355.1 | putative\_transmembrane\_efflux\_protein | BGC0000714 | Sisomicin\_biosynthetic\_gene\_cluster | 27 | 78.9661319073 | 111 | 3.3e-24 |
| ABB05093.1 | LipEx1 | BGC0001003 | Lipomycin\_biosynthetic\_gene\_cluster | 28 | 64.8841354724 | 107 | 6.2e-23 |
| ACB46467.1 | efflux\_permease | BGC0000082 | Kijanimicin\_biosynthetic\_gene\_cluster | 26 | 70.944741533 | 107 | 6.2e-23 |
| AAD32747.1 | Mct | BGC0000915 | Mitomycin\_biosynthetic\_gene\_cluster | 28 | 77.7183600713 | 106 | 1.1e-22 |
| AHL46729.1 | transporter\_protein | BGC0001179 | Rishirilide\_B\_biosynthetic\_gene\_cluster | 28 | 72.9055258467 | 106 | 1.4e-22 |
| AGI99475.1 | efflux\_permease | BGC0001004 | Lobophorin\_biosynthetic\_gene\_cluster | 25 | 73.6185383244 | 106 | 1.4e-22 |
| AGC09506.1 | LobA4 | BGC0001183 | Lobophorin\_biosynthetic\_gene\_cluster | 25 | 73.6185383244 | 105 | 1.8e-22 |
| ctg1\_orf530 |  | BGC0001199 | Akaeolide\_biosynthetic\_gene\_cluster | 27 | 77.3618538324 | 100 | 7.6e-21 |
| ACB37757.1 | putative\_multidrug\_export\_protein | BGC0000162 | Tetrocarcin\_A\_biosynthetic\_gene\_cluster | 25 | 71.1229946524 | 97 | 6.4e-20 |
| ADI71464.1 | putative\_transporter | BGC0000203 | BE-7585A\_biosynthetic\_gene\_cluster | 26 | 81.4616755793 | 91 | 3.5e-18 |
| ABS50496.1 | NapR6 | BGC0000652 | Napyradiomycin\_biosynthetic\_gene\_cluster | 22 | 80.2139037433 | 80 | 8.1e-15 |
| ABS50468.1 | NapR6 | BGC0001079 | Napyradiomycin\_biosynthetic\_gene\_cluster | 23 | 80.7486631016 | 79 | 1.1e-14 |
